# Advance on Resveratrol Application in Bone Regeneration: Progress and Perspectives for Use in Oral and Maxillofacial Surgery

**DOI:** 10.3390/biom9030094

**Published:** 2019-03-08

**Authors:** Denise Murgia, Rodolfo Mauceri, Giuseppina Campisi, Viviana De Caro

**Affiliations:** 1 Department of Biological, Chemical and Pharmaceutical Sciences and Technologies, University of Palermo, 90123 Palermo, Italy; denise.murgia@unipa.it; 2 Department of Surgical, Oncological and Oral Sciences, University of Palermo, 90127 Palermo, Italy; rodolfo.mauceri@unipa.it (R.M.); giuseppina.campisi@unipa.it (G.C.)

**Keywords:** Resveratrol, bone-regeneration, craniofacial tissue, alveolar bone loss, bone defect, resveratrol scaffold

## Abstract

The natural polyphenol Resveratrol (RSV) claims numerous positive effects on health due to the well documented biological effects demonstrating its potential as a disease-preventing agent and as adjuvant for treatment of a wide variety of chronic diseases. Since several studies, both in vitro and in vivo, have highlighted the protective bone aptitude of RSV both as promoter of osteoblasts’ proliferation and antagonist of osteoclasts’ differentiation, they could be interesting in view of applications in the field of dentistry and maxillofacial surgery. This review has brought together experimental findings on the use of RSV in the regeneration of bone tissue comprising also its application associated with scaffolds and non-transfusional hemocomponents.

## 1. Introduction

Resveratrol (trans-3,4′,5-trihydroxystilbene, RSV) is a naturally occurring polyphenol and stilbene derivative, obtained from food sources as such red wine and numerous fruits including grapes, peanuts, nuts, pistachios, cocoa, berries and some Asian medicinal herbs [1,2]. Resveratrol exists as cis- and trans-isomer. The preferred steric form is the trans-RSV that is relatively stable if protected from high pH values and light. Its synthesis in plants can be induced by microbial infections, ultraviolet (UV) radiations and exposure to ozone [3].

This compound has been studied for its several biologic effects and potential as a disease-preventing agent in prevention and treatment of a wide variety of chronic diseases [4].

Resveratrol is protective against oxidative cardiovascular disorders [5], being in part responsible for the “French paradox”, the phenomenon that explains a low incidence of cardiovascular diseases in a high-fat diet with contextually a moderate consumption of red wine [3]. This effect seems related to its antioxidant property [6], since it acts as scavenger of a number of free radicals already at low doses (10 μM), modulates lipid metabolism, protects low density lipoproteins (LDL) against oxidative and free radical damage and inhibits platelet activation and aggregation [7].

However, this action in vivo may be more attributable to its gene regulator effect than a direct scavenging action. Resveratrol downregulates the expression and activity of the oxidase inhibiting NADPH oxidase-mediated production of reactive oxygen species (ROS), stimulates mitochondria biogenesis reducing mitochondrial superoxide generation and upregulates the tetrahydrobiopterin-synthesizing enzyme guanosine triphosphate (GTP) cyclohydrolase I preventing superoxide production from uncoupled endothelial nitric oxide synthase. In addition, RSV increases the expression of various antioxidant enzymes [8].

Due to its action on gene regulation, RSV also possesses antiaging properties. It shows a strong capability of Sirtuin 1 (Sirt1) activation, an enzyme belonging to the families of sirtuins, known also for their antiaging action. Resveratrol is able to modulate the activity of numerous proteins through Sirt1 stimulation, as the peroxisome proliferator-activated receptor coactivator-1α (PGC-1 alpha), the forkhead family of transcription factors (FOXO) and protein kinase B (Akt). It can also modulate nuclear factor kappa B (NFκβ), which is an important sensor of toxic xenobiotics and oxidants, whose activation is a crucial cellular defence mechanism [9,10]. Moreover, its structural similarity to the synthetic oestrogen diethylstilbestrol lets it play a role as a phytoestrogen, making it able to blind to estrogenic receptors both in an agonist and antagonist manner, regulating the transcription of oestrogen-responsive genes, thus preventing age-related diseases [7]. Resveratrol modulates enzymes, pathways of inflammation mediators and induction of programmed cell death in activated immune cells, expressing also anti-inflammatory properties [3,11].

The cancer chemopreventive and anticancer activity of RSV is also known [1,12]. At 100 μM concentration, RSV seems to block the carcinogenesis principle stages of initiation [7], mainly due to induction of apoptosis via several pathways, such as mitogen-activated protein kinase (MAPK)- and p53-dependent mechanism [13], and to alteration of gene expression, bringing to a cancer initiation, promotion and progression [11].

Dental caries and periodontitis are very common diseases worldwide and are the main causes of tooth loss. As a consequence of tooth loss, resorption of the alveolar bone incurred as well as a reduction of the amount of available bone for the insertion of dental implant [14].

In maxillofacial reconstructive surgery, traditional bone regeneration techniques involve autologous, homologous, heterologous, or allogeneic grafts. Autologous bone grafts are considered the best option due to a low risk of immunogenicity or disease transmission but are limited because of inadequacy for repairing larger bone defects, donor-site morbidity, and potential wound-based infections, as well as the prolonged operative times.

The use of stem cells, as MSCs (mesenchymal stem cells), DPSCs (dental pulp stem cells) or adipose-derived stem cells, is one of the most interesting approaches, but it is still limited by their low accessibility, difficult collection, and poor long-term stability. Stem cells also need the combination with scaffolds or biomaterials to improve their efficacy and stability.

Some researchers have focused on investigating biomaterials embedding osteogenic growth factors (e.g., bone morphogenic proteins, vascular endothelial growth factor, fibroblast growth factor, insulin like growth factors) as a strategy to enhance bone regeneration and accelerate tissue healing. Despite their effectiveness in vitro, comparable results have been difficult to achieve in vivo, and it has been even more difficult to transfer their use into clinical protocols, considering also the large dosages needed. Moreover, the lability and the easy denaturation under extreme treatments of growth factors must be considered.

In terms of cost and efficacy, small molecules (<1000 Da), synthetically manufactured or isolated from natural sources, easy to manufacture, stable, processable, affordable and able to activate particular signaling pathways that lead to osteoblastic growth and differentiation without risk of host immune reaction could represent a valuable approach to overcoming limits of other strategies [15]. Considering that RSV closely matches the aforementioned characteristics and possesses interesting biochemical activities, the aim of this review was to collect all information about RSV effects on bone regeneration, in view of its future application in dentistry and maxillofacial surgery.

In vitro and in vivo experimental findings on bone tissue regeneration of RSV application alone or in association with new scaffolds and non-transfusional hemocomponents also open new perspectives on improving the clinical management of osteonecrosis of the jaw and the engraftment of dental implants.

## 2. Mechanisms of Bone Formation

Bone is the connective tissue having functions of protection for tissues and internal organs, mechanical support for muscles and depot for the systemic mineral homeostasis. Two types of bone could be distinguished, with different morphology and functionality.

The cortical component is the compact one, almost 80% of skeletal mass, located in diaphyseal regions of long bones and characterized by densely formed collagen fibrils surrounding a central channel for blood vessels, lymphatics and nerves, with mechanical and protective functions. Trabecular bone is located inside the cortical one and at the ends of long bones where the cortical component is thinner. It is a spongy tissue, made by a porous network of thin wires called *trabeculae* having metabolic functions [16].

This tissue has a dynamic nature; indeed, there is constant formation of new bone and dissolution of old, injured, or unnecessary bones for repair or for calcium release. The extracellular matrix is highly mineralized and it is produced by osteoblasts, bone cells responsible for forming new bone, derived from multipotent mesenchymal stem cells (MSCs) in the bone marrow and placed especially in the growing portions of bone, while the cells responsible for bone resorption, or breakdown, are the osteoclasts derived from hematopoietic progenitors (i.e., monocytes/macrophages) also in the bone marrow [17,18]. The remodelling process of bone exceeds in bone formation during the growth stages of childhood, adolescence and into young adulthood, while after this stage bone resorption exceeds bone formation, resulting in gradual and progressive bone loss [18].

Bone regeneration is a complex, well-orchestrated physiological process of bone formation, which can be seen during normal fracture healing, and is involved in continuous remodelling throughout adult life [19].

Bone turnover, and, consequently, bone mass can be influenced by several systemic factors, such as hormonal status, nutrition, physical inactivity, exposure to smoking, alcohol or particular drugs [17]. Due to the declining estrogen at menopause, women have a more rapid bone loss and a greater risk of osteoporosis than men [18,20].

The differentiation into osteoclasts is promoted by a bond between receptor activator of NF-κB ligand (Tnfsf11/RANKL) secreted by osteoblasts and RANK receptors located on the surface of hematopoietic cells [21]. During osteoclastogenesis, bone marrow macrophages (BMMs) differentiate into tartrate-resistant acid phosphatase (TRAP)-positive pre-osteoclasts, which then fuse with each other to form mature osteoclasts [22,23].

Macrophage colony-stimulating factor (M-CSF) and RANKL are two markers of osteoclasts formation [23], while osteoblast-secreted osteoprotegerin (OPG) acts as a decoy receptor inhibiting the interaction between RANKL and RANK [24].

Instead, osteoblast differentiation is stimulated by specific transcription factors since MSCs could differ into either adipocytes, chondrocytes or myoblasts [22,25]. The essential regulators of osteoblasts differentiation are runt-related transcription factor 2 (RUNX2) and Osterix downstream of RUNX2 that during the proliferation phase stimulate a morphological change from osteoprogenitors to preosteoblasts that start to synthetize bone matrix and alkaline phosphatase (ALP) [18]. Mature osteoblasts then regulate bone matrix mineralization and produce osteocalcin (OCN) that with ALP are used as clinical markers of bone formation [18].

Local and systemic factors as bone morphogenetic proteins, insulin-like growth factor (IGF), the canonical wingless (Wnt)/β-catenin signaling pathway, mechanical forces, estrogen and other hormones could influence the activity of transcription factors [18].

Mesenchymal stem cells could differ also in adipocytes; the main determinant is peroxisome proliferator-activated receptor γ2 (PPARγ2), but this differentiation could be decreased on behalf of osteoblasts with a Sirt1activation that acts inhibiting PPARγ2 [25]. Sirt1 acts deacetylating various transcription factors such as FOXO3A, a member of the multifunctional FOXO family. The FOXO3A functions as a direct and positive regulator of RUNX2 gene transcription, contributing to osteogenesis in MSCs [26].

In the context of the oral and maxillofacial area, the bone regeneration is primarily required for implant insertion in edentulous patients. Alveolar bone is the major support for the teeth and its resorption after tooth extraction or avulsion is very rapid probably due to disuse atrophy, decreased blood supply, localized inflammation or unfavorable prosthesis pressure [27,28].

Conventional bone regeneration techniques involve autologous grafts. Nevertheless, autogenous bone grafts present some drawbacks such as limited graft accessibility, prolonged operation time and donor site morbidity. Transplantation of cultured osteogenic cells represents an interesting new strategy to overcome these disadvantages. In particular, dental pulp stem cells (DPSCs) and periosteal stem cells seem to represent optimal alternatives to MSCs displaying high potential for differentiating into a variety of cell types, including osteocytes, suggesting their effective use in bone regeneration [29]. Dental pulp stem cells were self-expanding and differentiated into pre-osteoblasts able to self-maintain and renew. Moreover, in vitro they differentiated into osteoblasts producing living autologous fibrous bone tissue. Transplantation of this tissue in vivo led to the formation of lamellar bone with osteocytes without the need for scaffolding [30]. The periosteum also seems to be a source of stem cells useful for maxillary sinus augmentation for subsequent implantation. A clinical study proved that mandibular periosteum-derived osteoblasts in a suitable matrix showed to form lamellar bone within three months after transplantation in the edentulous posterior maxilla [31].

## 3. In Vitro Studies

The effects of RSV supplementation were tested on several cell lines. Tseng et al. worked on human embryonic stem cell–derived mesenchymal progenitors (EMPs), finding that RSV promotes spontaneous osteogenesis by upregulation of the expression of osteo-lineage genes RUNX2 and OCN but prevents adipocyte formation suppressing adipo-lineage genes PPARg2 and LEPTIN in adipogenic medium. Since EMPs could differentiate into osteogenic or adipogenic lines on stimulation in the appropriate medium, they found that RSV promotes the activation of RUNX2 gene transcription via Sirt1 and interacting with FOXO3A. Moreover, they observed that 5 mM RSV could enhance the action of ALP and osteogenic phenotype of calcium, increasing the osteogenesis of EMPs while the treatment with 10 mM RSV for six days prevented adipogenesis [26].

Daia et al. investigated in vitro effect of RSV on cell proliferation and osteoblastic maturation in human bone marrow-derived mesenchymal stem cells (HBMSCs) cultures treated with increasing concentrations of RSV (10^−8^–10^−4^ M). They observed an increase of (3H)-thymidine incorporation, ALP action and calcium deposition in HBMSCs, which meant an enhanced cell proliferation and osteoblast formation in a time- and dose-dependent manner up to 10^−5^ M. RSV induced a stimulation associated with estrogen receptor (ER) signaling and an increased ERK1/2 (extracellular signal–regulated kinases) activity that imply the rise of the osteogenic genes RUNX2/CBFA1 together with Osterix and OCN, the downstream genes of RUNX2/CBFA1 markers. Therefore, the osteogenic gene expressions were demonstrated by mechanisms concerning the ER-dependent MAPK pathway in HBMSC cultures [13].

The mechanism by RSV promotes osteoblastic differentiation from pluripotent mesenchymal cell line ST2 was evaluated by Zhou et al. in a study on factors involved in Wnt signalling, essential for bone formation. They worked on ST2 cells incubated in osteoblast-inducing medium treated for 14 days with RSV (0, 1, 5, 10, 50 or 100 μM). The Wnt signaling pathway stabilizes the β-catenin and its nuclear accumulation, increasing the expression of the critical genes for osteoblastic differentiation. The increased β-catenin activity was related to an increase in phosphorylation and inactivation of GSK-3β, even if this RSV-mediated stabilization depended on cells types and their growth conditions as well as the RSV doses applied. Surely, the treatment with RSV involves the phosphorylation of GSK-3β by the activation of ERK1/2 reducing its function but remained unknown what RSV directly acts on [32].

According to Ornsturp et al., RSV seems to inhibit proliferation and increase differentiation of osteoblasts independently of the inflammatory state. They studied if RSV affected proliferation and differentiation of HBMSCs and if RSV was able to counteract the negative effects of inflammation on cells development. They evaluated the effect of short (96 h) and long-term (21 days) RSV stimulation (25 µM), under both normal and inflammatory conditions (inducted by lipopolysaccharide, LPS). ALP, N-terminal propeptide of human type 1 collagen (P1NP) and osteoprotegerin (OPG) were considered as markers of osteoblast differentiation, lactate dehydrogenase (LDH) to assess cell death, whereas (interleukins, ILs) IL-6 and IL-8 as inflammatory markers. While short-term stimulation had no impact on proliferation and differentiation, the long-term one seemed to affect the osteoblastogenic potential of HBMSCs, given a three-fold increase in ALP and a petty one in P1NP and OPG, resulting in a strong decrease in proliferation and increase of differentiation of osteoblasts. About inflammatory state, RSV neither did affect IL-6 and IL-8 production in normal HBMSCs nor did significantly prevent inflammation in HBMSCs-LPS-treated [33].

Resveratrol seems to affect the mineralized matrix production of human and rat adipose derived stem cells (ADSCs). Dosier et al. evaluated the osteogenic potential of ADSCs in a three-dimensional (3D) culture environment like a polycaprolactone (PCL)/Collagen 3D Scaffold supplemented with different doses of RSV. For both rat and human cell constructs, the pre-treatment with 12.5 μM RSV involved a greater mineralized matrix at four weeks. Doses higher than 12.5 μM decreased the mineralized matrix that may mean a potentially cytotoxic effect. The greatest mineralized matrix was obtained with the dose of 6.25 μM RSV at the later time points. The overall authors’ results demonstrated that high RSV doses produce a faster differentiation and thus higher mineralized matrix levels at early time points, while low doses promote differentiation and retaining better cell viability, resulting in greater mineralized matrix at later time points. In general, a greater mineralization at all time-points was shown in ADSCs from rats compared to human ones. This may be related to differences in RSV metabolism, meaning that the mineralized matrix production from ADSCs depends on species as well as RSV dose [34]. These results suggest that the RSV dosage should be carefully considered and tailored in the perspective of its application in the field of bone tissue engineering. 

Table 1 shows an overview of the aforementioned results, where have been included cells type and RSV doses used together with bibliographic references.

## 4. In Vivo Studies

### 4.1. Resveratrol by Parenteral Administration

In oral and maxillofacial surgery, one of the most common surgical procedures is the tooth extraction, whose healing process, often uneventful, may occur inadequate in patients who are undergoing immunosuppressive therapy, such as CyclosporinA (CsA). To the aim of evaluating the potential benefic effects of antioxidant agents on bone health in these patients, Ozcan-Kucuk et al. studied the effect of intraperitoneal injection of RSV (10 μM/kg/die) on the healing of sockets for 14 or 28 days after tooth extractions in normal and CsA-treated rats. The bone healing process was evaluated and the results showed higher levels of OCN and osteopontin markers as well as an improved alveolar socket healing both in normal rats treated with RSV and in RSV-CsA-treated ones [35]. Regarding the role of RSV in the treatment of periodontitis, Bhattarai et al. studied in vitro the mechanisms of cellular responses of lipopolysaccharide (LPS)-stimulated human gingival fibroblasts (hGFs) treated with RSV. Moreover, they studied the effects of RSV treatment in vivo, by daily subcutaneous injection (5 mg/kg), in an experimental periodontitis model induced in rats by molar ligature infused with LPS. They observed that RSV treatment inhibits almost completely the outcomes of LPS stimulation, reducing ROS and inflammation-associated proteins levels, osteoclasts formation, and increasing heme oxygenase(HO)-1 gene expression via Nrf2-mediated signaling, thus protecting rats against alveolar bone loss, also demonstrated by micro-computed tomography CT analysis. These results demonstrated the RSV ability to inhibit inflammatory responses and stimulate antioxidant defence systems in periodontitis disease [36].

Melinjo seed extracts rich in RSV derivatives (MSE) also seem to counteract periodontal bone loss by modulation of an immune-inflammatory process involved in periodontal tissue destruction [37]. After periodontitis induction in mice (by ligatures in the gingival sulcus of the maxillary molar), an MSE standardized extract in a single dose of 0.004% (*w*/*w*) bodyweight was administrated by the intraperitoneal route. At time points of 17, 20, and 22 days, the animals were sacrificed and examined by morphometric and micro-CT image analyses that evidenced the reduction of hard periodontal tissue loss in MSE mice groups. Immunohistochemistry assays also demonstrated that, in gingival tissue, MSE utilization downregulated oxidative stress through Nrf2 pathway, modulated the expression levels of cytokine IL-1β, whereas IL-6, -17 and TNF-α, did not showed significant differences compared to the control. Moreover, MSE treatment inhibited M-CSF/sRANKL mediated osteoclast formation and downregulated osteoclast activity, reducing mainly osteoclastogenesis. These findings suggest that MSE treatment in periodontitis promotes periodontal bone regeneration by reduction of local oxidative damage and of osteoclast differentiation and/or activity. These findings suggest that MSE, or other RSV-like compounds, could provide a useful support for the management of periodontal inflammatory diseases [37].

Zhai et al. have demonstrated that RSV may improve local blood flow to bone in a rabbit model of steroid-induced osteonecrosis (ON) of the femoral head. Due to its anti-inflammatory effects, RSV protects the vascular endothelium, and reduces tissue hypoxia and thrombosis [38]. They evaluated the effect of 4 mg/kg/die RSV administered via i.p. for two weeks on preventing ON. Resveratrol dose-dependently inhibited endothelium and monocyte NF-κB transcription to reduce transcription factor (TF) production. Resveratrol was also shown to reduce LPS-, IL-1β-, and/or TNF-α-induced TF mRNA expression. In particular, in this study, TF expression was positive or strongly positive in the ON affected group, indicating that RSV reduced TF production and thrombosis formation [38].

Resveratrol seems to affect bone metabolism in ovariectomized rats. Lin et al. used bone mineral density as parameter to evaluate the effects of small, median, and high doses of RSV in ovariectomized rat groups compared to the estrogen-replacement control group. The results indicated that the daily RSV intake reduced bone turnover and prevented bone loss. Furthermore, the highest 45 mg/kg/die RSV dose seemed more effective in depressing bone resorption ovariectomy-induced than other doses [39].

### 4.2. Resveratrol by Oral Administration

Tamaki et al. investigated on the activity of RSV on progression of periodontitis demonstrating its capability of reducing alveolar bone resorption and systemic oxidative stress. They induced periodontitis in rats placing a ligature around the maxillary molars, also inducing the alveolar bone resorption; then, to the animals, 10 mg/kg/die of Melinjo RSV was orally administered. They observed in RSV treated group increased activation of the Sirt1/5′ adenosine monophosphate-activated protein kinase (AMPK) and Nrf2/antioxidant defense pathways, and inhibition of the NF-κB/MAPK pathway in inflamed gingival tissue. Moreover, melinjo RSV administration improved systemic levels of 8-OHdG (8-hydroxy-2′–deoxyguanosine), dityrosine, NOx, and nitrotyrosine, demonstrating that RSV intake could prevent the progression of periodontitis and alveolar bone loss [10].

The effects of RSV on bone healing and its influence on the gene expression of osteogenic markers were also investigated by Casarin et al. In the study, two calvarial defects were created and one screw-shaped titanium implant was inserted in the tibia of rats that were subjected to 10 mg/kg/die RSV administration by gavage for 30 days [40]. The histomorphometric analysis resulted in a reduced remaining defect in the RSV group. Furthermore, the torque force evaluation on removal of the implant highlighted that RSV treatment positively influenced the biomechanical retention of the titanium implant. Moreover, immunohistochemical results showed in an RSV group an increased expression of bone morphogenetic proteins (BMP)-2, BMP-7 and osteopontin (OPN) [40]. These results suggest that the long-term intake of RSV could represent a useful therapeutic approach for bone healing and in the rehabilitation of patients with dental implants.

In a study promoted by Pino et al., the influence of RSV was evaluated on the repair of bone calvarial defects in animals with induced diabetes mellitus (DM). The animals, subjected to critical-size calvarial defects, were treated by administering RSV (10 mg/kg/die) solution, insulin (INS) and both RSV and INS for 30 days. The histometric results indicated smaller residual calvarial defects in rats treated with RSV or RSV + INS that appeared in accordance to higher gene expression of BMP-2 observed in the RSV + INS group. No remarkable increase in BMP-2 gene expression was made evident in rats treated with RSV alone, demonstrating that only the combined RSV and INS administration was effective to markedly increase BMP-2 expression [41].

The potential anti-aging effect of RSV to counteract age-related bone loss was investigated by Tresguerres et al. Femora of old rats were analyzed in term of bone mass and biomechanical properties after dietary supplementation with 10 mg/kg/die RSV for 10 weeks. At the end of study bone volume, bone trabecular number and cortical thickness were improved in RSV treated animals, whereas the spacing between trabeculae was reduced. Moreover, all of the mechanical properties and bone microstructure were enhanced, except for the work to failure that has not been affected by RSV treatment, being dependent on the elasticity of collagen and mineral crystals orientation. An enhancement in the expression of Sirt1 and bone morphogenic protein (BMP) was also made evident, while neither plasma OCN nor CTX was modified by the treatment [42].

In addition, Durbin et al. investigated the effect of RSV supplementation on age-related bone loss considering mineralization of femora and tibiae. Old male rats were provided of 12.5 mg/kg bw/die RSV by oral gavage for 21 days before collecting tibia and femora. RSV supplementation prevented bone demineralization and bone microarchitecture loss. Furthermore, higher values of plasma OCN and ALP and lower of C-reactive protein were found in RSV-treated rats. The authors supposed that the anti-inflammatory activity of RSV, able to suppress the production of various cytokines and other pro-inflammatory mediators, is the main mechanism responsible for an increase in osteoblast bone formation and attenuation in age-related skeletal disuse as demineralization, microstructure deterioration, and bone loss [43].

Even if RSV seems to have a positive action on old rats, the potential effects of RSV supplementation during the rapid postnatal growth period and in late adulthood (early ageing) seem to have negative effects on bone microarchitecture and metabolism. A study suggests that 2.5 mg/kg/die RSV supplementation for five weeks in young rats (4-week-old) does not significantly affect bone structure and volume during the rapid growth phase. Nevertheless, RSV doses of 20 mg/kg/die for three months could potentially have negative effects on skeletons during early ageing in 6-month-old rats [44]. Despite the fact that RSV treatment did not affect bone micro-architecture in the young animals, in the early ageing rats, a trend of reduction in trabecular bone volume was observed together with a reduction of Osterix and OCN expression and an increased expression of adipogenesis-related genes. Furthermore, serum levels of bone resorption marker CTX-1 were significantly raised in the RSV group, while no changes of Sirt1 and ALP markers were observed [44].

Resveratrol effect on bone healing and its influence on the gene expression of bone-related markers in rats exposed to cigarette smoke was evaluated by Franck et al. Wistar rats, subjected to calvarial defects, were treated with RSV *plus* smoke exposure (SMK + RSV). Resveratrol (10 mg/kg/die) was administered for 30 days following surgery, while smoke inhalation was started seven days before surgery and continued for 37 days. Histomorphometric analysis highlighted in the SMK + RSV group the reduction of remaining calvarial defects respect to placebo-treated rats exposed to smoke. Immunohistochemical assays showed that RSV therapy in rats exposed to smoke inhalation decreased RANKL/OPG expression and Dkk1 levels, upregulating the gene expression of bone remodeling markers (RUNX2), optimizing the repair of critical-sized bone defects [45].

The effect of RSV treatment on bone was evaluated also in obese men suffering from Metabolic syndrome (MetS), a disease associated with low-grade inflammation, which may harm bones [46]. In the randomized, double-blinded, placebo-controlled trial, the subjects were treated with 1000 mg RSV (RSVhigh) or 150 mg RSV (RSVlow) daily for 16 weeks and bone mineral density, bone mineral content and biochemistry parameters were monitored. The researchers concluded that RSV acts in a dose-dependent manner and promotes bone formation or mineralization directly without mediation of anti-inflammatory process [46]. 

### 4.3. Resveratrol by Local Administration

In a study by Uysal et al., the effects of local RSV application on bone formation in rats were evaluated in response to expansion of the interpremaxillary upper incisors [47]. A single-dose of 10 µmol/kg RSV was injected into the interpremaxillary suture 24 h after appliance placement, evaluating histomorphometrically the bone formation in the suture. In particular, the area and the perimeter of new bone, Feret’s diameter, the percentage of new bone to non-ossified tissue and the number of osteoblasts were measured. All of these parameters were significantly improved in the RSV-group [47]. These results could encourage the local application of RSV to promote fractured bone repair and bone formation or in the reconstruction of bone defects, including the management of osteoporosis.

A summary of the outcomes of aforementioned RSV in vivo studies, including experimental design and bibliographic references, is provided in Table 2.

## 5. Innovative Scaffolds Loaded with Resveratrol

Resveratrol is well known for its poor aqueous solubility and rapid metabolism and excretion as sulfate and monoglucuronide derivatives. The poor bioavailability is a major drawback of the drug. Different approaches have been used to improve solubility, stability and bioavailability of RSV by formulation of drug delivery systems (DDSs) in the form of scaffolds able to release RSV at the site of bone loss to improve and optimize its efficacy.

A scaffold loaded with RSV was prepared grafting RSV to polyacrylic acid (PAA, 1000 Da), PAA-RSV, and then incorporating this macromolecular drug into atelocollagen (Coll) hydrogels (Coll/PAA-RSV) [48]. The scaffold was tested in vitro both on chondrocytes and BMSCs and in vivo on rabbits with osteochondral defects by an implant. In vitro results showed that the scaffold could support the growth and maintain the morphology and phenotype of chondrocytes and BMSCs and it is able to protect them against reactive oxygen species, demonstrating an excellent cytocompatibility. After implantation of Coll/PAA-RSV scaffold for two, four and six weeks on rabbits, the inflammatory related genes IL-1β, matrix metalloproteinases-13, cyclooxygenase (COX)-2 were downregulated while bone and cartilage related genes SOX-9, aggrecan, Coll II and Coll I were upregulated resulting in an anti-inflammatory functionality. Moreover, after 12 weeks, the osteochondral defects completely disappeared and the neo-cartilage was well integrated with surrounding tissue and subchondral bone. The distribution of Coll II and glycosaminoglycans in the regenerated cartilage was confirmed by immunohistochemical and glycosaminoglycan staining [48].

Resveratrol was grafted to the surface of porous poly-ε-caprolactone (PCL) by a covalent linkage with the carboxylic groups of acrylic acid (AA) to produce a scaffold with osteogenic effect, tested on BMSCs and in the rat calvarial defect model [49]. The ALP activity in stromal cells and the mineralization of the cell–scaffold composites resulted in being increased by the presence of RSV. In vivo osteoinductive effects were evaluated by implanting a scaffold in rat calvarial defects. After eight weeks, the increased bone regenerating capacity of RSV-PLC scaffold was highlighted by X-ray and histological analyses [49].

A 3-D porous PLC scaffold was formulated with RSV-loaded albumin nanoparticles (RNP) to form a PCL-RNP-RSV composite scaffold with improved osteoconductive, osteoinductive, and osteogenic capacities [50]. Resveratrol was released from PCL-RNP in a sustained-manner for 12 days, until a total release of 64%. In vitro experiments on HBMSCs showed a significant increase in proliferation, ALP increase and mineralization induced by PCL-RNP-RSV compared with the PCL scaffold. PCL-RNP-RSV scaffold was also cytocompatible [50].

A chitosan-poly-ε-caprolactone composite nanofibrous scaffold for wound dressing able to simultaneously deliver ferulic acid and RSV was designed by Poornima and Korrapati [51]. In vitro cytocompatibility and hemocompatibility was evaluated, whereas, in vivo on Wistar rats, the full thickness skin wound healing was studied. The nanofibers were able to maintain a sustained release of actives, showed compatibility with keratinocytes and enhanced healing of skin wounds in vivo [51].

Since RSV could promote osteogenesis and inhibit adipogenesis in mesenchymal cell lines, electrospun drug-eluting fibers loaded with RSV were designed to be used in the regenerative dentistry for the post-extraction preservation of the alveolar socket [52]. Uniform defect-free membranes based on fibers of PCL or poly(lactic) acid (PLA) containing RSV were produced and the kinetics of RSV release as well as their osteoinductive capacity on DPSCs were evaluated. An initial burst followed by a slow release was shown by a PCL-RSV membrane, while PLA-RSV presented a much slower and continuous release over time. In vitro experiments on DPSCs highlighted that the RSV concentration range released from the two nanofibers influenced osteoblast and osteoclast differentiation differently. In particular, both materials were able to promote DPSCs differentiation into osteoblast-like phenotypes, increasing gene expression of the osteogenic markers and inducing calcium deposition after 28 days of incubation. However, the RANKL-induced osteoclast differentiation was inhibited by PLA-RSV and only this reduced TRAP activity and catephsin K gene expression. The PLA-RSV could represent a useful scaffold for dentistry applications able to limit the physiological remodeling process that could affect correct implant placement [52].

The combination of collagen scaffold containing RSV with human adipose stem cells (hASCs) for craniofacial tissue-engineering applications both in vitro and in vivo was evaluated by Wang et al. The collagen/RSV biocomposite scaffold used in hASCs differentiations demonstrated the complete biocompatibility of RSV and its role in the high differentiation rates of stem cells and in calcium deposition. The in vivo results on surgically induced oral mucosal defects indicated that the great effectiveness of scaffolds in promoting epidermal wound healing. Furthermore, on rats with critical-sized calvarial defects, the micro-CT analysis showed that hASCs-cultured collagen/RSV scaffold implanted had a better bone mineralization and defect regeneration than hASCs-collagen scaffold without RSV. The overall results suggested that collagen scaffolds loading RSV were more effective than empty ones at enhancing epithelial and osteogenic differentiation of hASCs [53].

## 6. Resveratrol with Platelet-Rich Plasma and Other Hemocomponents

In the last few years, a new guided bone regeneration procedure was introduced in oral surgery, based on the use of non-transfusional hemocomponents, as platelet-rich plasma (PRP) [54], even if, in literature, there has still been little produced about the application of RSV together with them.

Surely, one of the most popular beneficial effects of RSV is the prevention of atherosclerosis and coronary heart disease. Wang et al. investigated RSV influence on aggregation of platelets obtained from healthy, normotensive male volunteers and in hypercholesterolemic rabbits, using Born’s method to measure the platelet aggregation rate [55]. Both in vitro and in vivo results suggest that RSV can inhibit platelet aggregation and this could be correlated with its cardioprotective effect. In vitro effects were evaluated using platelet-rich plasma (PRP) from healthy subjects. It was observed that the aggregation of platelets was significantly inhibited by 10–1000 µM RSV, in a concentration-dependent manner. In in vivo experiments, 4 mg/kg/day RSV administration showed in hypercholesterolemic rabbits, an inhibition of ADP-induced platelet aggregation but had no effect on serum lipid levels [55].

Medication-related osteonecrosis of the jaw (MRONJ) is an adverse drug reaction that consists of progressive bone destruction in the maxillofacial area of patients exposed to the treatment with drugs associated with the risk of ONJ, in the absence of a previous radiation treatment [56].

Usually, MRONJ is related to long-term treatment with bisphosphonates, a first line treatment for metastatic bone cancer and osteoporosis.

Recently, many authors investigated the application of autologous platelet concentrates for the prevention and treatment of MRONJ [57,58]. For the therapy in osteoclast-mediated bone loss, bisphosphonates are used even if the long-term treatment is associated with pathological conditions including osteonecrosis of the jaw, specifically named Bisphosphonates-related osteonecrosis of the jaw (BRONJ) [59].

In vitro effects of concentrated growth factors (CGF) and/or RSV on proliferation and differentiation of human osteoblasts, treated or not with bisphosphonates, were investigated by Borsari et al. [59]. Platelet concentration (also named CGF) was prepared centrifuging blood samples with a special machine that, at the end of the process, formed tree fractions: the platelet poor plasma (PPP) in the upper layer, free red blood cells (RBC) in the lower layer and CGF in the middle layer, used for the study. Resveratrol at 10 μM concentration was used. The results, obtained by both MTT (3-[4,5-dimethylthiazol-2-yl]-2,5-diphenyltetrazolium bromide) assay and the evaluation of some osteogenic markers using ELISA (enzyme-linked immunosorbent assay) and immunohistochemical analysis, showed that in vitro osteoblast proliferation and differentiation is promoted by both CGF and RSV, which had a protective role on osteoblasts treated with bisphosphonates, especially zoledronate. This activity is also improved by co-treatment, making these findings promising for the clinical management of BRONJ [59].

## 7. Conclusions

Bioengineering strategies to promote the bone regeneration in oral and maxillofacial areas has developed over the last decade as a result of the progresses in the fields of stem cell biology, polymer chemistry, and clinical procedures. Recently, there has also been a surge in unconventional and innovative approaches to restore bone defects or prevent them by treating periodontal diseases.

Resveratrol, due to its immunomodulatory effects and ability to decrease levels of some proinflammatory cytokines, seems to fit perfectly with the scenery of these new biotechnologies, as a further element to improve regenerative progression of alveolar bone.

According to what detailed was in this review, RSV administration could represent a valid therapeutic approach for bone healing processes and in the rehabilitation of edentulous patients with dental implants, showing a wide-ranging potential also in the treatment of oral diseases related to oxidative stress. Moreover, RSV supplementation, enhancing growth, proliferation, and differentiation of MSCs, DPSCs and ADSCs, potentially offers a number of advantages over the use of stem cells alone, scaffolds or hemocomponents alone, in terms of healing of periodontitis, restoration of bone defects and biomechanical retention of titanium implants.

Finally, RSV application alone or in association with new scaffolds and non-transfusional hemocomponents open new perspectives to improve the clinical management of a wide range of oral diseases including osteonecrosis of the jaw related to long-term treatment with bisphosphonates.

To date, the clinical use of RSV has been limited mainly by its poor solubility in physiological fluids, resulting in a low bioavailability. The most recent literature reports new nanotechnologies [60] able to overcome these drawbacks, prospecting in a short future its use in clinical practice for a wide range of disorders, due to its anti-inflammatory, anti-oxidant, and anti-tumor properties.

## Figures and Tables

**Table 1:** Summary of Resveratrol’s in vitro studies.

Authors	Cell Type	RSV Doses	Results
Tseng et al. 2011 [26]	EMPs	5, 10 µM	Promoted osteogenesis (5 mM)
			adipogenesis Prevented (10 mM)
			↑ RUNX2
			↑ OCN
			↑ ALP
			↓ PPARg2
			↓ Leptin
Dai et al. 2007 [13]	HBMSCs	0.01, 0.1, 1.0, 10 µM	Promoted osteogenesis
			↑ RUNX2
			↑ OCN
			↑ ALP
			↑ Calcium deposition
			↑ ERK1/2
Zhou et al. 2009 [32]	ST2 cells	1, 5, 10, 50, 100 μM	↑ ERK1/2
			↑ β-catenin
Ornstrup et al. 2016 [33]	HBMSCs	25 μM	↑ osteoblastogenic
			↑ ALP
Dosier et al. 2012 [34]	rat ADSCs human ADSCs	6.25, 12.5, 25 μM	↑ Mineralized matrix production (6.25 μM)

EMP: human embryonic stem cell–derived mesenchymal progenitors; HBMSC: human bone marrow-derived mesenchymal stem cells; RUNX2: runt-related transcription factor 2; OCN: osteocalcin; ALP: alkaline phosphatase; ERK1/2: extracellular signal–regulated kinases 1/2; ST2: pluripotent mesenchymal cell line; ADSCs: human and rat adipose derived stem cells.

**Table 2:** Summary of Resveratrol’s in vivo studies.

Authors	Animal Model	Induced Disease	Administration Route	Biochemistry Results	Morphometric Outcomes
Ozcan-Kucuk et al. 2018 [35]	Normal Rats CsA treated rat	Tooth extraction	Intraperitoneal injection 10 µmol/kg daily	↑ OCN in normal and CsA-rats ↑ Osteopontin	↑ New bone formation ↑ Epithelization ↓ Inflammation
Bhattarai et al. 2016 [36]	Rats	ligature-induced Periodontitis	Subcutaneous injection 5 mg/kg daily	↑ HO-1 induction via Nrf2-mediated signalling ↓ Inflammation-related proteins ↓ Formation of osteoclasts ↓ Production of circulating ROS	↑ BMD ↑ BV/TV (%)
Ikeda et al. 2018 [37]	Mice	ligature-induced Periodontitis	Intraperitoneal injection 0.001% *w*/*w* only once	↓ Oxidative stress ↓ M-CSF/sRANKL mediated osteoclast formation ↓ Osteoclast activity	↓ ABL
Zhai et al. 2016 [38]	Rabbits	steroid-induced osteonecrosis of the femoral head	Intraperitoneal injection 4 mg/kg Every day for 2 weeks	↓ NF-κB transcription ↓ TF production ↓ LPS ↓ Interleukin-1β- ↓ TNF-α-induced TF mRNA expression	↓ ON incidence
Lin et al. 2005 [39]	Rats	Ovariectomized	Parenteral 45 mg/kg per day		↑ BMD ↓ Bone turnover Reverse previous bone loss
Tamaki et al. 2014 [10]	Rats	ligature-induced periodontitis	Orally 10 mg/kg/die	↑ Sirt1/AMPK activation ↑ Nrf2/antioxidant defence pathways ↓ NF-κB/MAPK pathway ↓ Systemic 8-OHdG, dityrosine, ↓ NOx, nitrotyrosine ↓ IL-1β, IL-6, and TNF-α	↓ ABL
Casarin et al. 2014 [40]	Rats	Calvarial defects and titanium implant	By gavage 10 mg/kg/die for 30 days	↑ BMP-2 ↑ BMP-7 ↑ OPN BSP, OPG, RANKL equal to control	↓ Critical-size calvarial defects ↑ counter-torque force for implant removal
Pino et al. 2017 [41]	Diabetic rats	Critical-sized calvarial defects	Orally solution 10 mg/kg for 30 days	↑ BMP-2 ↑ BMP-2 ↑ Osterix	↓ Critical-size calvarial defects
Tresguerres et al. 2014 [42]	Rats	Age-related bone loss	Orally 10 mg/kg per day for 10 weeks	↑ BMP ↑ Sirt1 CTX equal to controlOCN equal to control	↑ BV/TV ↑ trabecular number ↑ Cortical thickness ↓ Space between trabeculae ↑ Mechanical properties
Durbin et al. 2014 [43]	Rats	Age-related bone loss	Oral gavage 12.5 mg/kg bw/day for 21 days	↑ ALP ↓ C-reactive protein ↑ Plasma OCN ↑ Osteoblast bone formation	↓ BMD, BMC loss ↓ BV/TV loss ↑ Phosphorus content ↑ trabecular connectivity ↑ trabecular number
Lee et al. 2014 [44]	4-week-old Wistar rats 6-month-old Wistar rats		Orally 5 mg/kg/day for 5 weeks 20 mg/kg/day for 3 months	In young rats: In ageing rats: ↓ Osterix↓Sirt1 gene expression ↓ OCN ↑ Adipogenesis-Regulatory Genes ↑ CTX-1 Sirt1 equal to control ALP equal to control	In young rats: BV/TV equal to control In ageing rats: ↓ BV/TV
Franck et al. 2018 [45]	Rats	Calvarial defects together with smoke exposure	Orally 10 mg/kg daily for 30 days	↓ RANKL/OPG ↓ Dkk1 ↑ RUNX2	No differences to control group
Ornstrup et al. 2014 [46]	Obese Men with MetS		1000 mg (RSV_high_) or 150 mg (RSV_low_) daily for 16 weeks	↑ Bone Alkaline Phosphatase P1NP equal to control OCN in equal to control OPG equal to control CTX equal to control S-NTx equal to control	↑ vBMD in RSV_high_ ↑ aBMD in RSV_high_ ↑ BMC in RSV_high_
Uysal et al. 2011 [47]	Rats	Bone formation in response to expansion of upper incisors	Local 10 µmol/kg single-dose		↑ new bone area ↑ new bone perimeter ↑ Feret’s diameter ↑ Newly formed bone (%) ↑ Number of osteoblast

BMD = Bone mineral density; BV/TV = bone volume/tissue volume (%); ABL = alveolar bone loss; BMA = Bone mineral area; BMC = bone mineral content; aBMD = Areal bone mineral density; BMC = bone mineral content; vBMD = Volumetric bone mineral density; BSP = bone sialoprotein; S-NTx = serum concentration of type I collagen N-telopeptide.
